# PFKFB4 promotes lung adenocarcinoma progression via phosphorylating and activating transcriptional coactivator SRC-2

**DOI:** 10.1186/s12890-021-01420-x

**Published:** 2021-02-16

**Authors:** Jiguang Meng, Xuxin Chen, Zhihai Han

**Affiliations:** Department of Respiratory Medicine, Sixth Medical Center of Chinese People’s Liberation Army General Hospital, Beijing, 100048 China

**Keywords:** PFKFB4, Lung adenocarcinoma (LUAD), SRC-2, Phosphorylation

## Abstract

**Background:**

To investigate the role and its potential mechanism of 6-phosphofructo-2-kinase/fructose-2,6-bisphosphatase 4 (PFKFB4) in lung adenocarcinoma.

**Methods:**

Co-immunoprecipitation was performed to analyze the interaction between PFKFB4 and SRC-2. Western blot was used to investigate the phosphorylation of steroid receptor coactivator-2 (SRC-2) on the condition that PFKFB4 was knockdown. Transcriptome sequencing was performed to find the downstream target of SRC-2. Cell Counting Kit-8 (CCK-8) assay, transwell assay and transwell-matrigel assay were used to examine the proliferation, migration and invasion abilities in A549 and NCI-H1975 cells with different treatment.

**Results:**

In our study we found that PFKFB4 was overexpressed in lung adenocarcinoma associated with SRC family protein and had an interaction with SRC-2. PFKFB4 could phosphorylate SRC-2 at Ser487, which altered SRC-2 transcriptional activity. Functionally, PFKFB4 promoted lung adenocarcinoma cells proliferation, migration and invasion by phosphorylating SRC-2. Furthermore, we identified that CARM1 was transcriptionally regulated by SRC-2 and involved in PFKFB4-SRC-2 axis on lung adenocarcinoma progression.

**Conclusions:**

Our research reveal that PFKFB4 promotes lung adenocarcinoma cells proliferation, migration and invasion via enhancing phosphorylated SRC-2-mediated CARM1 expression.

## Background

6-phosphofructo-2-kinase/fructose-2, 6-biphosphatase-4 (PFKFB4) is a bi-function enzyme [1, 2]. On one hand, PFKFB4 synthesizes fructose 2,6-bisphosphate (F2,6-BP) to stimulate glycolysis, playing important roles in metabolic processes [3]. On the other hand, PFKFB4 acts as a phosphatase to hydrolyze F2,6-BP into fructose-6-phosphate (F6P) and inorganic phosphate (Pi), functioning as a protein kinase to phosphorylate protein substrates. The dual function of PFKFB4 makes it involved in multiple biological processes such as cell cycle regulation, cell proliferation, autophagy, and transcriptional regulation [2].

PFKFB4 has been reported to be involved in a variety of cancers including human bladder cancer, gastric cancer, colon cancer, breast cancer and other malignant tumors [2, 4–6]. It was found that PFKFB4 was overexpressed under hypoxia in gastric and pancreatic cancer cells and contributed to cancer cells proliferation and survival [5]. Recent studies have shown that metabolic enzyme PFKFB4 plays important roles in malignant breast tumors. High expression of PFKFB4 was associated with poor prognosis of operable breast cancer [7]. PFKFB4 phosphorylated and activated transcriptional coactivator SRC-3 to promote aggressive breast cancers [2]. Epithelial and endothelial tyrosine kinase (Etk) interaction with PFKFB4 modulated chemoresistance of small-cell lung cancer (SCLC) by regulating autophagy [8]. Studies indicated that PFKFB4 was essential for tumors progression. Mechanistically, during neural crest late specification, AKT signaling mediates PFKFB4 function, which was essential for premigratory and migratory neural crest formation [9]. PFKFB4 was critical for the survival of acute monocytic leukemia (AMoL) cells, serving as a downstream target of MLL through the putative E2F6 binding site in the promoter of the PFKFB4 gene, which might be a potent therapeutic target in AMoL [10]. However, the exact function and regulatory mechanism of PFKFB4 in lung adenocarcinoma (LUAD) is less understood.

The enzyme co-activator associated arginine methyltransferase 1 (CARM1), also known as protein arginine methyltransferase 4 (PRMT 4), has been reported to play critical roles in embryonic development and multiple cancers [11–14]. Early Studies have shown that CARM1 is often highly expressed in human cancers, including breast, ovarian, prostate, colorectal cancer and so on [15–17]. Recent studies have reported that CARM1 was related to tumor progression and metastasis. CARM1 and USP7-dependent LSD1 stabilization promotes invasion and metastasis of breast cancer cells [18]. CARM1 is essential for myeloid leukemogenesis and knockdown of CARM1 impairs cell-cycle progression and promotes myeloid differentiation [19]. It was reported that CARM1 could promote non-small cell lung cancer progression by upregulating CCNE2 expression [20]. These findings suggest that CARM1 functions as an oncogene in human cancers.

In present study we found that PFKFB4 and SRC protein family was aberrantly overexpressed at mRNA level analyzed from the ENCORI Pan-Cancer Analysis Platform. Further studies showed that PFKFB4 interacted with SRC-2 and phosphorylated SRC-2 at Ser487. Phosphorylated SRC-2 transcriptionally up-regulated CARM1 expression. Functionally, PFKFB4 promoted LUAD cell proliferation, cell migration and invasion by regulating phosphorylated SRC-2 mediated CARM1 expression. These findings revealed a pivotal role of PFKFB4 in LUAD progression by phosphorylation activation of SRC-2, thus upregulating CARM1 expression.

## Methods

### Bioinformatics analysis

The expression profile of PFKFB4 and SRC family at mRNA level in lung adenocarcinoma tissues was analyzed from UALCAN Platform (http://ualcan.path.uab.edu/index.html) which is a comprehensive, user-friendly, and interactive web resource for analyzing cancer OMICS data.

### RNA isolation, sequencing and expression analysis

A549 cells which were acquired from Shanghai Chinese Academy of Sciences Cell Bank (Shanghai, China) were transfected with SRC-2 #1 siRNA, SRC-2 #2 siRNA or control siRNA, respectively. Cells were harvested after transfection 72 h. Total RNA was extracted from cells with Trizol reagent (Invitrogen, Carlsbad, CA, USA). RNA purity was analyzed using NanoDrop (Quawell UV–visible spectrophotometer) and Agilent 2100 Bioanalyzer (Santa Clara, CA, USA). Afterwards, transcriptome sequencing was performed on the Illumina HiSeq™ 2500 (NxGenBio Life Sciences, New Delhi) platform. Clean reads were got by filtering for low-quality then assembled. The contigs were organized based on the overlap regions to get transcript sequences. Fold change with an absolute value of log_2_ ratio ≥ 5 was set as the threshold to obtain the transcripts of which expression was significant difference.

### Cell culture and cell transfection

NCI-H1975, A549 and 293 T cell lines were acquired from Shanghai Chinese Academy of Sciences Cell Bank (Shanghai, China). The lung adenocarcinoma cell line NCI-H1975 was cultured in RPMI1640 medium (Invitrogen, USA) and A549 was cultured in F12K (Invitrogen, USA) supplemented with 10% fetal bovine serum (FBS, Gibco, Grand Island, NY, USA) and 100 μg/ml streptomycin and 100 IU/ml penicillin (Invitrogen, USA). 293 T cells were cultured in DMEM medium (Invitrogen, USA) supplemented with 10% FBS and 1% penicillin/streptomycin. All cells were cultured at 37 °C with 5% CO_2_. The transfection experiments were performed using Lipofectamine 2000 Reagent (Invitrogen, USA). The wildtype plasmids were purchased from OriGene (Rockville, MD, USA). SRC-2 S487A was constructed by site-directed mutation. The sequences of shRNA against PFKFB4 and siRNAs against SRC-2 were shown in Table 1.

**Table 1:** Sequences for shRNA and siRNA

shRNA	Sequences
PFKFB4	CCGGGACGTGGTCAAGACCTACAAACTCGAGTTTGTAGGTCTTGACCACGTCTTTTTG
siRNA	Sequences (5′–3′)
SRC-2 #1	Guide: UUCUAUAUAUUUAUUUUCCUG
	Passenger: GGAAAAUAAAUAUAUAGAAGA
SRC-2 #2	Guide: ACGAUUACGUUUUUCAGUGUU
	Passenger: CACUGAAAAACGUAAUCGUGA

### Lentivirus-mediated generation of PFKFB4-knockdown cells

293 T cells were co-transfected with pLKO shRNA constructs, PAX2, MD2 helper plasmids using Lipofectamine 2000 Reagent (Invitrogen, USA). Following transfection, the lentivirus supernatants were collected. A549 and NCI-H1975 were infected for 3 days and selected in the presence of puromycin (1 μg/ml) for at least one week.

### Cell Counting Kit (CCK-8) assay

Cell proliferation was measured using cell counting kit-8 (CCK-8; Dojindo Molecular Technologies, Rockville, MD, USA). Cells were transfected as indicated. Cells were seeded into 96-well plates at a density of 3 × 10^3^ cells/well. CCK-8 reagent was added into the wells at indicated time points and incubated at 37 °C for 2 h. The absorbance was measured at 450 nm in a microplate reader (Promega, Madison, WI, USA).

### Transwell migration/invasion assay

For migration analysis, cells transfected after 48 h were seeded into the top compartment of the Transwell chamber (8-μm pore size; BD Biosciences, San Jose, CA, USA) in a 24-well plate at a proper density. DMEM medium containing 10% FBS was added to the bottom chamber. When cultured for 24 h, cells on the upper surface of the membrane were scraped. Cells which went through the membrane were fixed with 4% paraformaldehyde and stained with DAPI. The images were captured using the microscope. For invasion analysis, the upper Transwell chamber was coated with matrigel. The following steps were conducted as the migration assay. Cells going through the matrigel were collected and counted. Each experiment was performed in triplicate.

### Immunoprecipitation

Cells were harvested with lysis buffer with protease and phosphatase inhibitor cocktail (Millipore). Lysates were precleared with Protein A/G Agarose beads (Pierce) at 4 °C for 1 h, after washed then incubated with antibodies against Myc (Cell signaling Technology, #2276), or HA (Abcam, ab18181), or PFKFB4 (GeneX Health) respectively at 4 °C overnight. Preblocked agarose beads added to the lysates mixture were incubated at 4 °C for another 1 h. The immunocomplexes were eluted for western blot analysis.

### Western blot

Proteins were extracted using lysis buffer and separated by 10% SDS-PAGE (Invitrogen) and blotted onto Polyvinylidene Fluoride (PVDF) membranes. Membranes were incubated with primary antibodies at 4 °C overnight. Then membranes were incubated with the secondary antibodies at room temperature for one hour. Proteins were detected using the enhanced chemiluminescence (ECL) kit (Millipore, Burlington, MA, USA). The antibodies against SRC-2 phospho Ser469, phospho Ser487, phospho Ser493, phospho Ser499 and phospho Ser736 were prepared from GeneX Health Co,. Ltd.

### Quantitative real-time PCR (qRT-PCR)

Total RNA was extracted from cells using Trizol reagent (Invitrogen). RNA was reversely transcribed into cDNA using PrimeScript RT Master Mix (TaKaRa, Dalian, China). The cDNA was subjected on Real-time polymerase chain reaction (PCR) analysis using the SYBR qPCR-detection-Kit (TaKaRA, Dalian, China) on the Real-time PCR System (ABI7500, ABI, Oyster Bay, NY, USA). Glyceraldehyde-3-phosphate dehydrogenase (GAPDH) was used as the internal control. The sequences of the primers used for RT-PCR were listed in Table 2.

**Table 2:** Primers sequences for qRT-PCR

Genes	Primer sequences
CARM1	F: 5′-TTGATGTTGGCTGTGGCTCTGG-3′
	R: 5′-ATGGGCTCCGAGATGATGATGTCC-3′
MMP24	F:5′-CTCTGGCCAGTGCTCACC-3′
	R:5′- CCCATAATTCCCTGCCCCAC-3′
MAP7	F:5′-CAGTGCGAAGCGAAACAGC-3′
	R:5′-TGTTTCTCCCGTTCCTCACG-3′
ZFP91	F:5′-GCATGGGACAGCTCAGACTT-3′
	R:5′-GTTGGACGATCGGGTTGGAA-3′
ANKH	F:5′-CATCGGAGTGGACTTTGCCT-3′
	R:5′-GATCAGGAAGGGCATACCCAG-3′
LASP1	F:5′-CCCAAGCAGTCCTTCACCAT-3′
	R:5′-AGGAATCACAAGCTGTCGCA-3′
ATG2A	F:5′-TGCCAATCTGCTGTGAGAGG-3′
	R:5′-ACGCACTCACGGAGCTTAAA-3′
HSPA8	F:5′-CCCCATCATCACCAAGCTGT-3′
	R:5′-CTCCACCACCAGGAAATCCC-3′
ULK1	F:5′-CCTGAGGAGACCCTCATGGA-3′
	R:5′-CACAGCTTGCACTTGGTGAC-3′
CCNG2	F:5′-AACAAAAACAAGGGGCTCGG-3′
	R:5′-ATCATTCTCCGGGGTAGCCT-3′
GAPDH	F: 5′-CTCTGATTTGGTCGTATTGGG-3′
	R: 5′-TGGAAGATGGTGATGGGATT-3′

### Statistical analysis

All data were analyzed using GraphPad Prism5 software (GraphPad Software, San Diego, CA). Results were presented as the mean ± SD. Statistical significance was determined by the Student’s t-test.

## Results

### SRC-2 was identified to be an interacting protein with PFKFB4

**Fig. 1:**
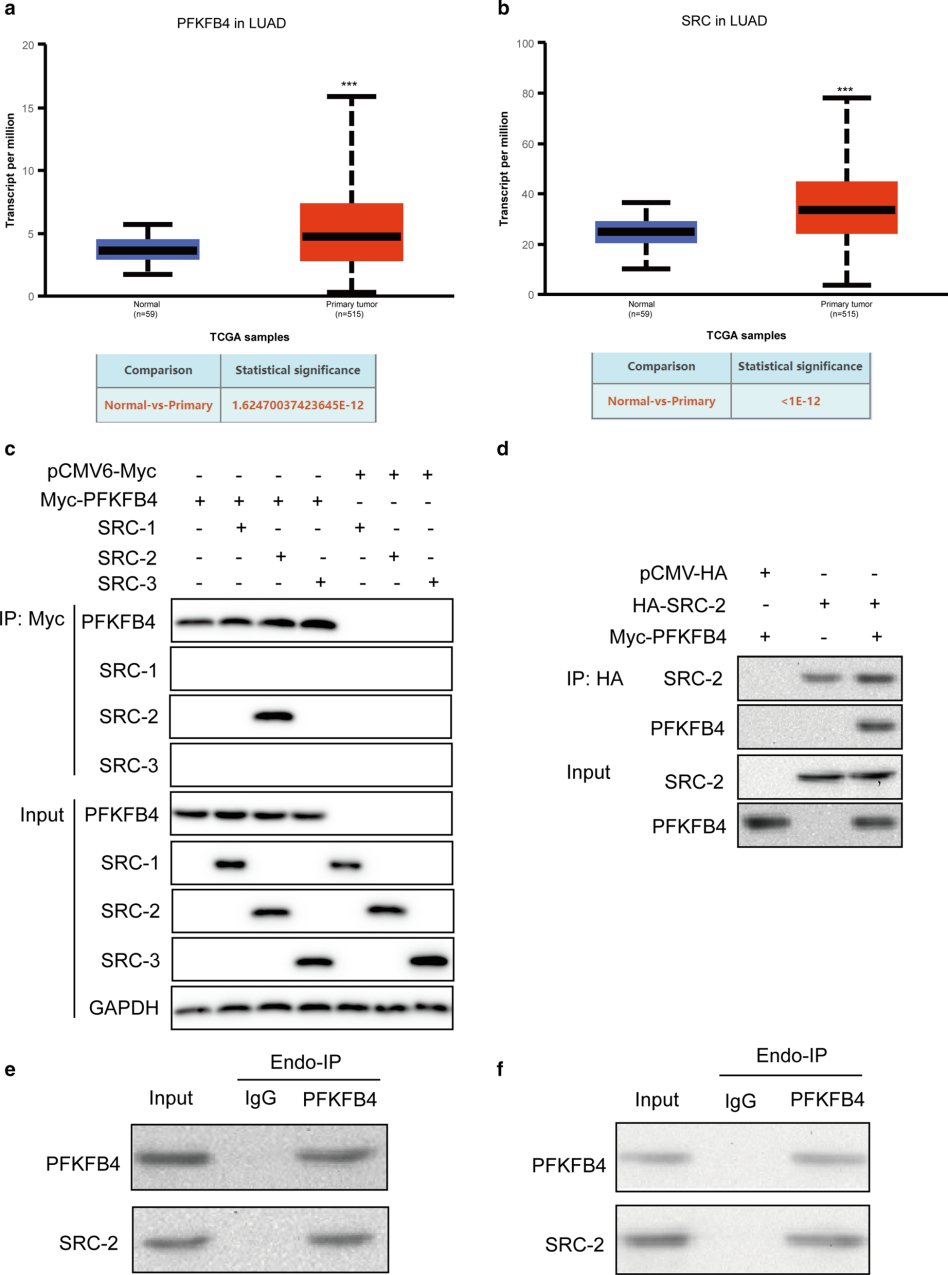
PFKFB4 interacted with SRC-2 in lung adenocarcinoma cells. **a**, **b** Expression of PFKFB4 and SRC family was analyzed among 515 primary tumors and 59 normal samples in lung adenocarcinoma (LUAD) via UALCAN Platform (http://ualcan.path.uab.edu/index.html). **c** 293 T cells expressing myc-PFKFB4 was transfected with three SRC family members (SRC-1, SRC-2 or SRC-3) respectively. Myc–PFKFB4 was immunoprecipitated followed by western blot to detect the interaction status of SRC family proteins. pCMV6-Myc was used as a negative control. **d** 293 T cells expressing HA-SRC-2 was transfected with empty vector or myc-PFKFB4. HA-SRC-2 was immunoprecipitated followed by western blot to detect the interaction with PFKFB4. pCMV6-HA was used as a negative control. **e**, **f** Endogenous immunoprecipitation was performed in A549 and NCI-H1975 cells with or without anti-PFKFB4 antibody. The interacted endogenous SRC-2 was detected by western blot. IgG was used as a negative control

It has been reported that metabolic enzyme PFKFB4 could promote breast cancer progression by activating the oncogenic steroid receptor coactivator-3 (SRC-3) [2]. Since we found that both PFKFB4 and SRC protein had a higher expression at mRNA level in 515 LUAD samples compared to 59 normal tissues (Fig. 1a, b) from UALCAN Platform (http://ualcan.path.uab.edu/index.html), we wondered whether PFKFB4 also had an interaction with SRC family proteins in LUAD. We next performed co-immunoprecipitation using Myc-tag in 293 T cells transfected with myc-PFKFB4 together with SRC-1, SRC-2 or SRC-3 respectively. We observed that PFKFB4 only interacted with SRC-2 but not SRC-1 or SRC-3 (Fig. 1c). Next, we validated the direct interaction between SRC-2 and PFKFB4 by co-precipitating SRC-2 with HA-tag in PFKFB4-expressing 293 T cells (Fig. 1d). Furthermore, endogenous co-immunoprecipitation results also confirmed that PFKFB4 interacted with SRC-2 in lung adenocarcinoma cells A549 and NCI-H1975 (Fig. 1e, f).

### PFKFB4 phosphorylated SRC-2 at Ser487

**Fig. 2:**
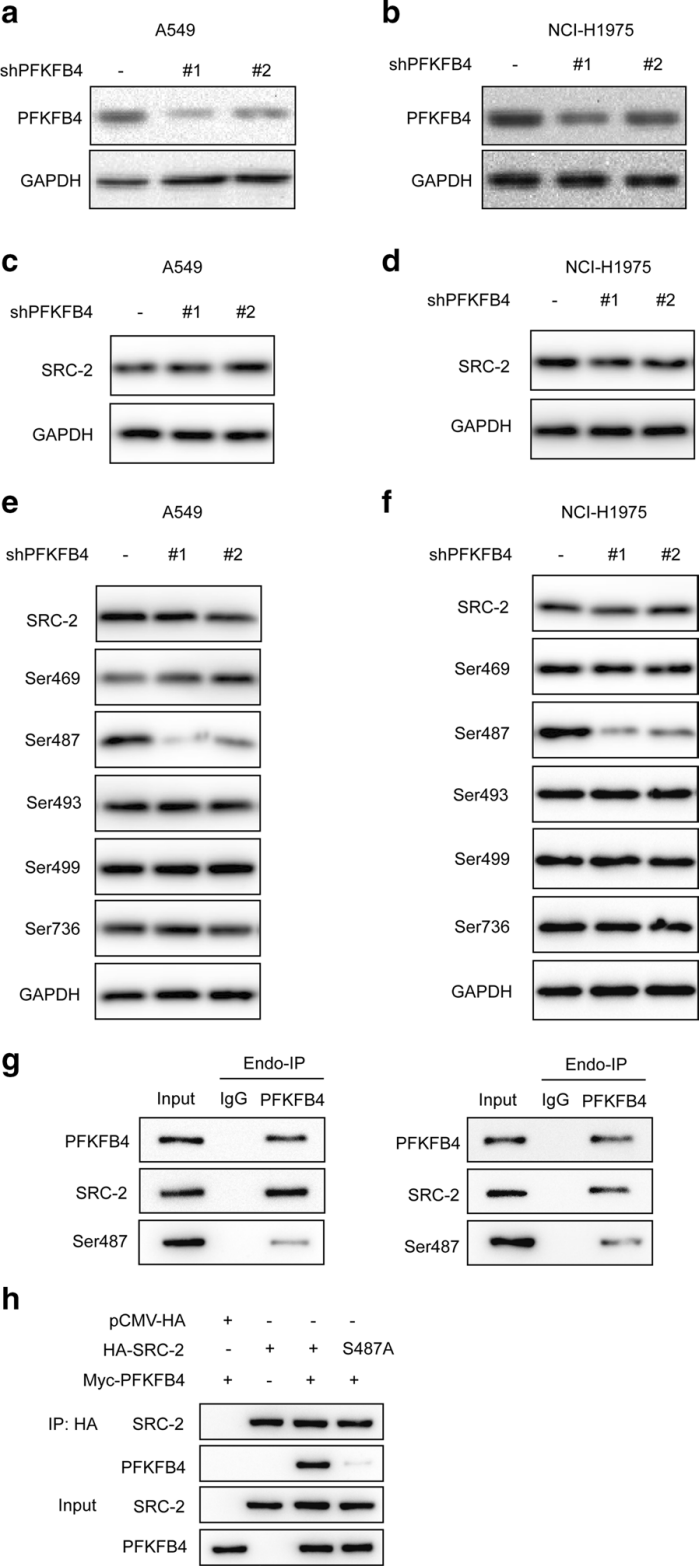
PFKFB4 phosphorylated SRC-2 at Ser487. **a**, **b** The knockdown efficiency of shPFKFB4 (#1 and #2) in A549 and NCI-H1975 was determined by western blot. GAPDH served as an internal control. **c**, **d** SRC-2 expression was detected by western blot in A549 and NCI-H1975 expressing shNC and shPFKFB4 (#1 and #2). GAPDH served as an internal control. **e**, **f** The phosphorylation of SRC-2 at Ser469, Ser487, Ser493, Ser499 and Ser736 in A549 and NCI-H1975 expressing shPFKFB4 (#1 and #2) was detected by western blot. **g** Endogenous immunoprecipitation was performed in A549 and NCI-H1975 cells with or without anti-PFKFB4 antibody. The phosphorylated SRC-2 at Ser487 was detected by western blot. IgG was used as a negative control. (H) Co-immunoprecipitation between PFKFB4 and SRC-2 WT or the phosphor-deficient SRC-2 mutant (SRC-2 S487A) was performed in A549 cells

To investigate the interactive relationship between PFKFB4 and SRC-2, firstly we selected two kinds of validated PFKFB4 shRNAs. The PFKFB4-knockdown stable cell lines were infected by lentivirus. The silencing efficiency was detected as shown in Fig. 2a, b. Both shPFKFB4s significantly reduced PFKFB4 expression compared to negative control (sh-NC) in A549 and NCI-H1975 cell lines. The shPFKFB4 #1 group had a better knockdown efficiency in comparison to the shPFKFB4 #2 group and would be used in subsequent functional experiments. We observed that PFKFB4-knockdown did not change SRC-2 expression at protein level (Fig. 2c, d). What’s more, the phosphorylated levels of SRC-2 at Ser487, of which the site was associated with transcription activity, was remarkably diminished when PFKFB4 expression was reduced (Fig. 2e, f). At the same time, the other phosphorylation sites of SRC-2 at Ser469, Ser493, Ser499, Ser736 had no change, indicating that PFKFB4 interacted with SRC-2 and phosphorylated SRC-2 at Ser487. Furthermore, we observed that the phosphorylated SRC-2 at S487 could also be detected in precipitant from SRC-2 immunoprecipitation of lung adenocarcinoma cell A549 and NCI-H1975 (Fig. 2g). Consistently, compared to those with overexpression of SRC-2 WT, co-IP of PFKFB4 would precipitate lower level of SRC-2 in cells with overexpression of the phosphor-deficient SRC-2 mutant (SRC-2 S487A) (Fig. 2h). Taken together, these results indicated that PFKFB4 could interact and phosphorylate SRC-2 at Ser487.

### CARM1 was the downstream target of SRC-2

**Fig. 3:**
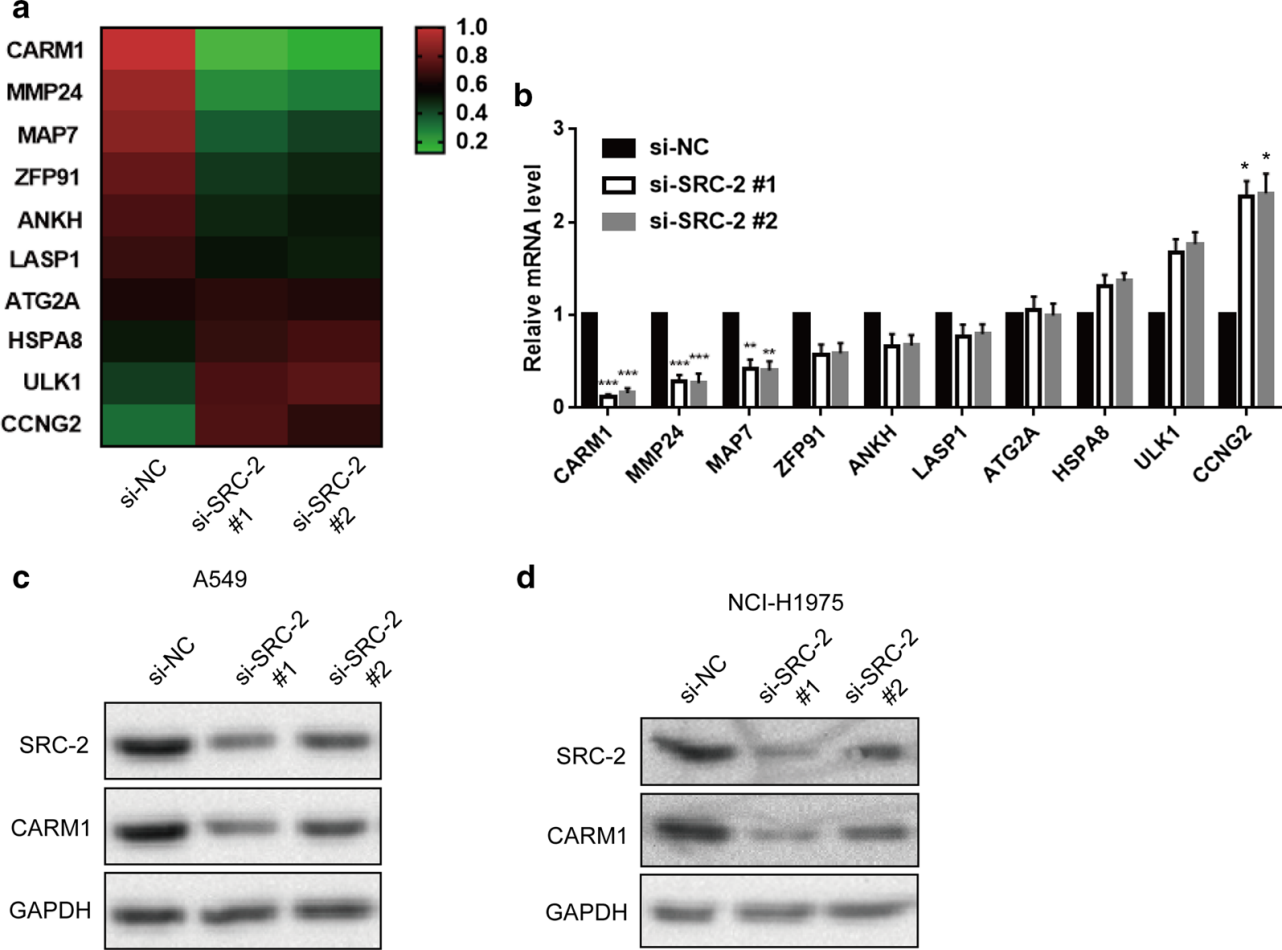
SRC-2 regulated CARM1 expression in lung adenocarcinoma cells. **a** Transcriptional analysis was performed in A549 expressing si-NC and si-SRC-2 #1/#2. There was an independent biological replicates in transcriptome sequencing. **b** CARM1 expression at mRNA level in A549 cells expressing si-NC and si-SRC-2 #1/#2 was detected by qRT-PCR. Relative mRNA expression was normalized to GAPDH. Data were represented as the mean ± SD. *P < 0.05, **P < 0.01, ***P < 0.001. All experiments were performed in triple. **c**, **d** CARM1 expression at protein level was detected by western blot in A549 and NCI-H1975 cells expressing si-NC and si-SRC-2 #1/#2

To identify the potential mechanism of PFKFB4-SRC-2 in lung adenocarcinoma, we performed a transcriptome sequencing using A549 cells transfected with si-SRC-2 #1 and si-NC. CARM1 expression was the most downregulated gene among these genes (Fig. 3a). The screening result was further validated by qRT-PCR. CARM1 expression at mRNA level was the most decreased among the downregulated genes in si-SRC-2 #1-transfected cells compared to control (Fig. 3b). Western blot showed that CARM1 expression at protein level was also reduced when SRC-2 was knocked down (Fig. 3c, d). The CARM1 protein level was decreased much more obviously in si-SRC-2 #1-transfected A549 and NCI-H1975 cells as si-SRC-2 #1 had a higher silencing efficiency compared to si-SRC-2 #2.

### PFKFB4 promoted lung adenocarcinoma cells proliferation, migration and invasion by phosphorylating SRC-2 at Ser487

**Fig. 4:**
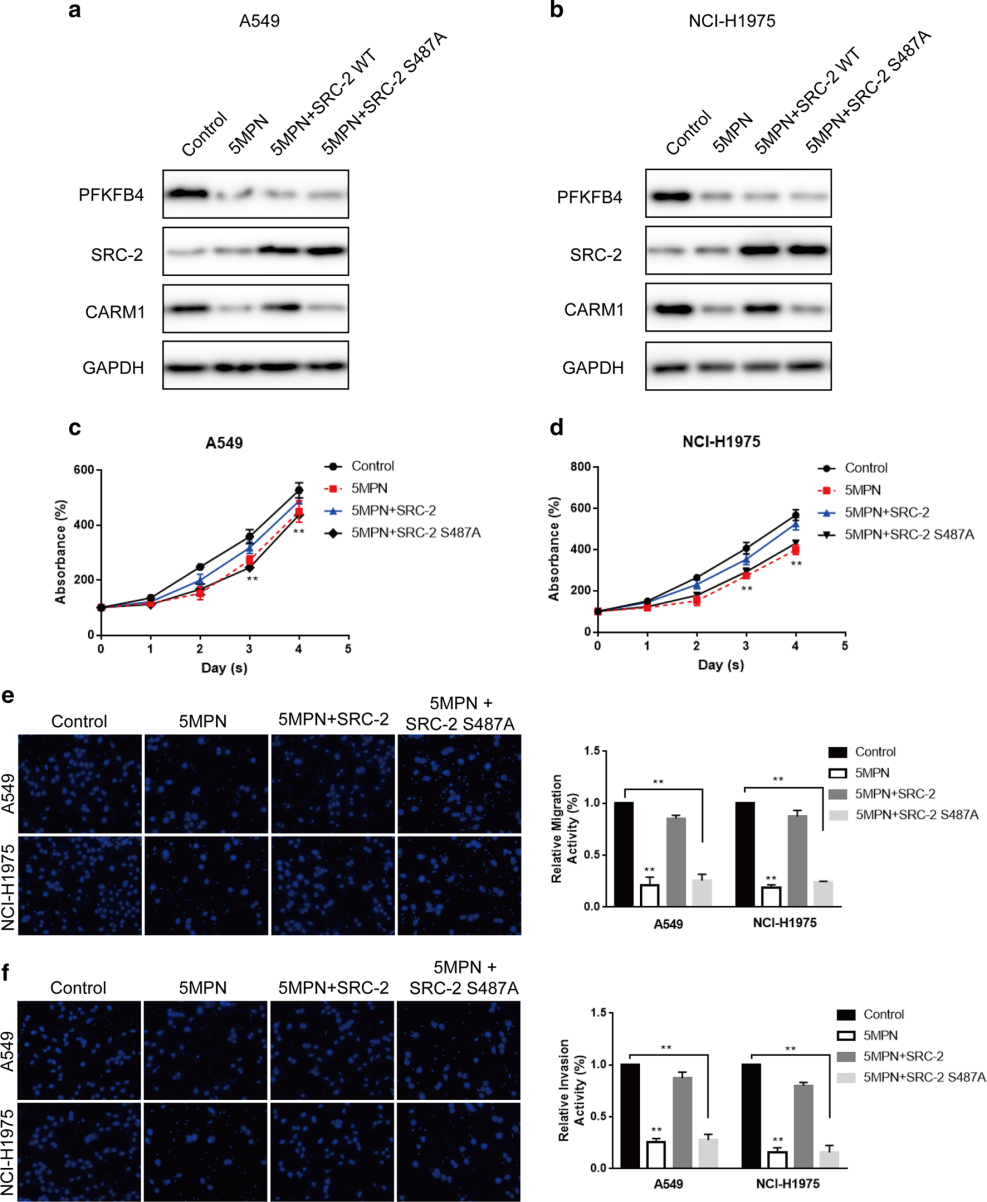
PFKFB4 promoted lung adenocarcinoma cells proliferation, migration and invasion via phosphorylating SRC-2 at Ser487. **a**, **b** A549 and NCI-H1975 were treated with DMSO or 10 μM 5MPN and/or transfected with pCMV vector and/or SRC-2 WT/S487A. The expression of PFKFB4, SRC-2 and CARM1 at protein level was examined by western blot. **c**, **d** CCK-8 assay was performed among A549 and NCI-H1975 treated with DMSO or 10 μM 5MPN and/or transfected with pCMV vector and/or SRC-2 WT/S487A. All experiments are performed in triple. **e**, **f** Transwell and Transwell-matrigel assay were performed among A549 and NCI-H1975 treated with DMSO or 10 μM 5MPN and/or transfected with pCMV vector and/or SRC-2 WT/S487A, respectively. Representative images are captured with the magnification of × 200. **P < 0.01. All experiments are performed in triple

To further explore the functional effects of the phosphorylation of SRC-2 at Ser487 in lung adenocarcinoma progression, we constructed a phosphorylation-deficient SRC-2 mutant plasmid (SRC-2 S487A). We transfected SRC-2 wildtype (SRC-2 WT) or SRC-2 S487A in 5MPN-treated (PFKFB4 inhibitor) A549 and NCI-H1975 cells. Western blot analysis showed that SRC-2 expression at protein level was not changed in 5MPN-treated A549 and NCI-H1975 cells while highly expressed with overexpression of SRC-2 WT or SRC-2 S487A. What’s more, CARM1 expression was decreased with 5MPN treatment and could only be rescued by SRC-2 WT overexpression but not SRC-2 S487A overexpression (Fig. 4a, b). The results suggested that PFKFB4 phosphorylated SRC-2 at Ser487 and activated its transcriptional activity so that CARM1 was up-regulated by phosphorylated SRC-2 at Ser487. Cell Counting Kit-8 (CCK-8) analysis showed that the decreased cell proliferation induced by 5MPN treatment in A549 and NCI-H1975 cells could be rescued by co-overexpression of SRC-2 WT but not SRC-2 S487A (Fig. 4c, d). Furthermore, overexpression of SRC-2 WT but not SRC-S487A in LUAD cells with low expression of PFKFB4 also restored the suppressed migration and invasion ability induced by PFKFB4 downregulation (Fig. 4e, f). In accord with the above results, we observed the similar functional effects by knocking down PFKFB4 with shRNA (Additional file 1: Figure S1). Above all, the results indicated that PFKFB4 promoted LUAD cells proliferation, migration and invasion dependent on the phosphorylation of SRC-2 at Ser487.

### SRC-2 co-activated CARM1 to drive LUAD cells proliferation, migration and invasion

**Fig. 5:**
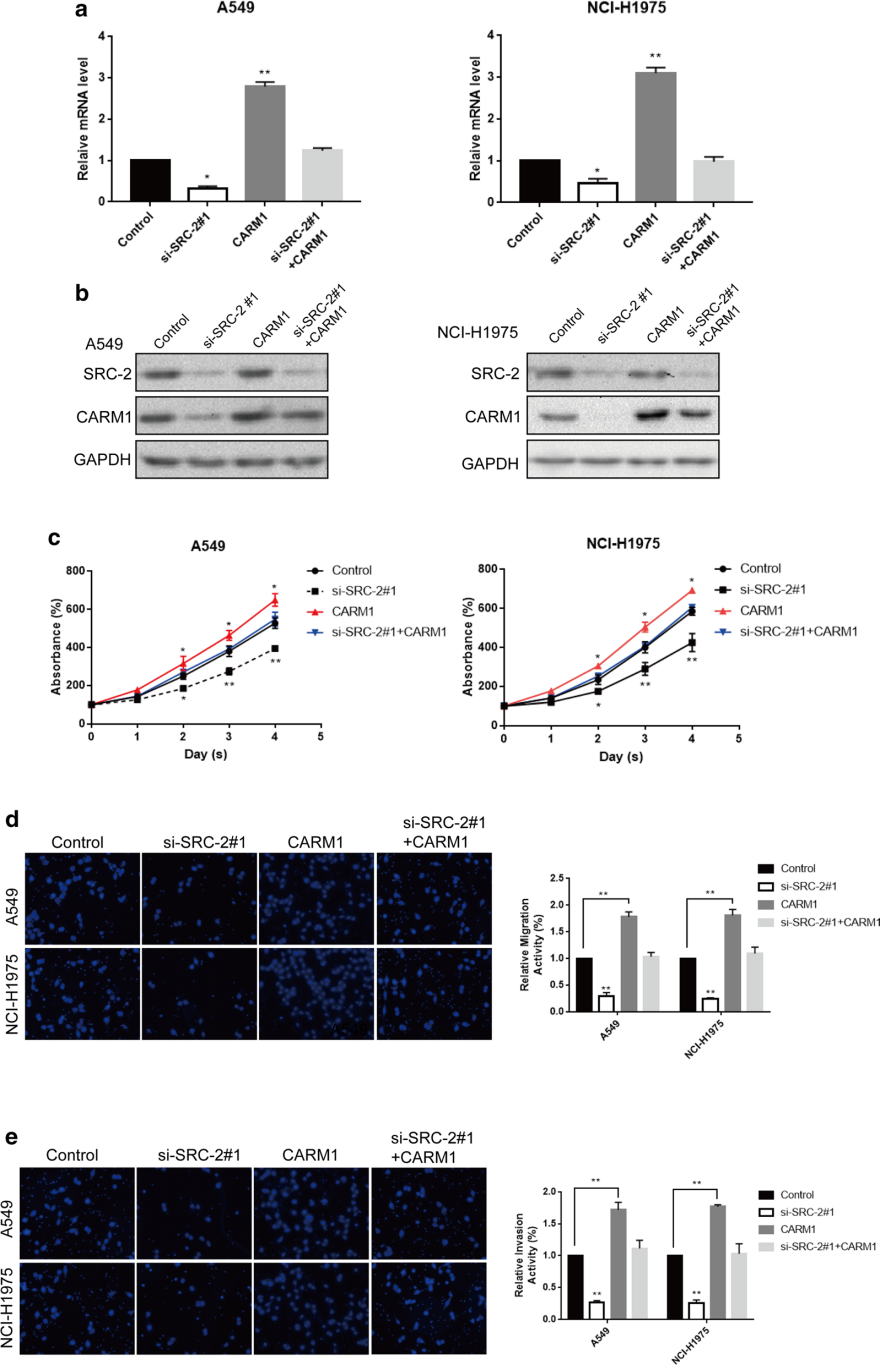
CARM1 was essential for lung adenocarcinoma cells proliferation, migration and invasion. **a** CARM1 expression at mRNA level was examined in A549 and NCI-H1975 transfected with si-SRC-2#1, CARM1, si-SRC-2#1 + CARM1 or control by qRT-PCR. *P < 0.05; **P < 0.01. All experiments are performed in triple. **b** CARM1 expression at protein level was examined in A549 and NCI-H1975 transfected with si-SRC-2#1, CARM1, si-SRC-2#1 + CARM1 or control by western blot. **c** CCK-8 assay was performed among A549 and NCI-H1975 transfected with si-SRC-2#1, CARM1, si-SRC-2#1 + CARM1 or control. *P < 0.05; **P < 0.01. All experiments are performed in triple. **d**, **e** Transwell and Transwell-matrigel assay were performed among A549 and NCI-H1975 transfected with si-SRC-2#1, CARM1, si-SRC-2#1 + CARM1 or control. Representative images are captured with the magnification of × 200. **P < 0.01. All experiments are performed in triple

To understand the precise molecular mechanism of PFKFB4-SRC-2-drived lung adenocarcinoma progression, we overexpressed CARM1 in si-SRC-2 #1-transfected LUAD cells, of which SRC-2 had been mostly knockdown. We observed that the decreased CARM1 expression at mRNA and protein levels in si-SRC-2#1-transfected A549 and NCI-H1975 cells could be rescued by co-overexpression of CARM1 (Fig. 5a, b). CCK-8 assays showed that overexpression of CARM1 in SRC-2-knockdown LUAD cells could blocked the suppressed A549 and NCI-H1975 cells proliferation resulted from si-SRC-2 #1 transfection (Fig. 5c). In addition, the blockade of migration and invasion abilities induced by SRC-2 knockdown could be restored by CARM1 co-overexpression (Fig. 5d, e). These data revealed that CARM1 was transcriptional up-regulated by phosphorylated SRC-2 at Ser487, enhancing lung adenocarcinoma cells proliferation, migration and invasion.

### PFKFB4 promoted lung adenocarcinoma cells proliferation through SCR-2/CARM1 axis

**Fig. 6:**
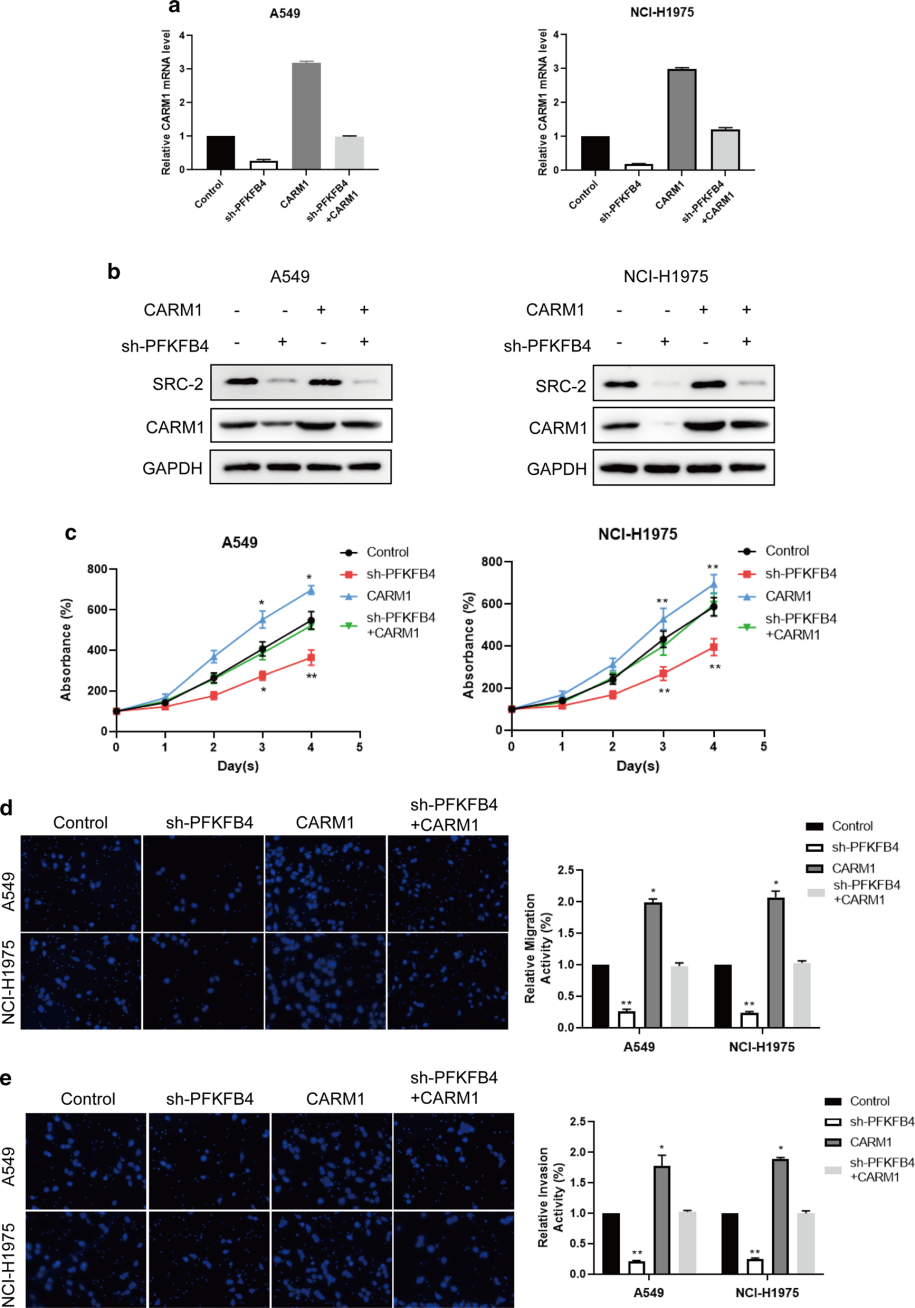
PFKFB4 promoted lung adenocarcinoma cells proliferation, migration and invasion by regulating SRC-2/CARM1. **a** CARM1 expression at mRNA level was examined in A549 and NCI-H1975 transfected with sh-PFKFB4, CARM1, sh-PFKFB4 + CARM1 or control by qRT-PCR. *P < 0.05; **P < 0.01. All experiments are performed in triple. **b** CARM1 expression at protein level was examined in A549 and NCI-H1975 transfected with sh-PFKFB4, CARM1, sh-PFKFB4 + CARM1 or control by western blot. **c** CCK-8 assay was performed among A549 and NCI-H1975 transfected with sh-PFKFB4, CARM1, sh-PFKFB4 + CARM1 or control. *P < 0.05; **P < 0.01. All experiments are performed in triple. **d**, **e** Transwell and Transwell-matrigel assay were performed among A549 and NCI-H1975 transfected with sh-PFKFB4, CARM1, sh-PFKFB4 + CARM1 or control. Representative images are captured with the magnification of × 200. **P < 0.01. All experiments are performed in triple

The above results indicated that suppressed effects resulted from SRC-2 knockdown could be mediated by CARM1 overexpression, but it was unclear that whether the inhibitory effects induced by PFKFB4 knockdown could be rescue by CARM1. Thus, we overexpressed CARM1 in PFKFB4-knockdown cells and detected the functional effects. The results showed that the decreased CARM1 expression at mRNA and protein levels led to by PFKFB4 knockdown could be rescued by CARM1 overexpression in A549 and NCI-H1975 cells (Fig. 6a, b). Moreover, the suppressed proliferation ability of PFKFB4 knockdown in lung adenocarcinoma cells was reversed upon overexpression of CARM1 in PFKFB4-knockdown A549 and NCI-H1975 cells (Fig. 6c). Furthermore, we also observed that CARM1 overexpression rescued the inhibitory effect of PFKFB4 knockdown on cell migration and invasion of A549 and NCI-H1975 cells (Fig. 6d, e). Taken together, these data showed that overexpression of CARM1 could abolish the suppressed effects induced by PFKFB4 knockdown, which suggested that PFKFB4 promoted lung adenocarcinoma cells proliferation through SCR-2/CARM1 axis.

## Conclusions

To sum up, our results demonstrate that PFKFB4 promoted LUAD progression dependent on SRC-2 transcriptional activity. Phosphorylation of SRC-2 at S487 increased its transcriptional activity and upregulated CARM1 expression, thus promoting lung adenocarcinoma cells proliferation, migration and invasion. Our findings uncover the function and molecular mechanism of PFKFB4-SRC-2 signaling in LUAD progression, providing a new sight into therapy and treatment on LUAD.

## Discussion

PFKFB4 is one of isoenzymes of PFK2 (i.e., PFKFB1, PFKFB2, PFKFB3, and PFKFB4) which is a well-known bifunctional enzyme owning kinase and phosphatase activities [21]. Previous studies have showed that PFKFB4 acted as a key sugar-phosphate metabolite to stimulate glycolysis in various biological processes [3]. In recent years, PFKFB4, acting as a protein kinase, has been found to play a vital role in many cancers including glioblastoma [22], bladder cancer [23], gastric cancer and pancreatic cancer [24], breast cancer [2] and so on. Studies showed that loss of PFKFB4 in brain cancer stem-like cells promoted cell death while inhibited lactate secretion, which was essential for the maintain of the brain cancer stem-cells stemness [22]. PFKFB4 was required for peroxisome proliferator-activating receptor γ (PPARγ)-stimulated glycolysis in hepatocellular carcinoma [25]. However, the importance and exact mechanism of PFKFB4 in lung adenocarcinoma progression is lack of research. In our study, we found that PFKFB4 was overexpressed in lung adenocarcinoma tissues and cell lines (A549 and NCI-H1975) and further revealed the regulatory mechanism of PFKFB4 in LUAD cell proliferation, migration and invasion.

Interaction of PFKFB4 with steroid receptor coactivator-3 (SRC-3) protein has been found as a key regulatory mechanism in breast tumor. Phosphorylated SRC-3 transcriptionally upregulates transketolase expression to promote breast tumor growth and metastasis to the lung [2]. SRC-3, as a transcriptional coregulatory is deregulated in many tumors, which belongs to the SRCs (SRC-1, SRC-2 and SRC-3) family. Studies have shown that SRCs family especially SRC-2 and SRC-3 is involved in normal biological processes and carcinogenesis. For example, SRC-3 activation promoted tumor growth of pancreatic ductal adenocarcinoma [26]. SRC-2-mediated coactivation of anti-tumorigenic target genes suppresses MYC-induced liver cancer [27]. SRC-2 inhibition severely attenuated the survival, growth, and metastasis of prostate cancer [28]. Based on the previous studies, we wondered whether PFKFB4 regulated LUAD progression via SRCs family protein. We found that the expression of SRC family protein was also elevated in LUAD tumor tissues via the ENCORI Pan-Cancer Analysis Platform. Furthermore, we identified that SRC-2 interacted with PFKFB4 in vitro and in vivo. PFKFB4 phosphorylated SRC-2 at Ser487, of which site was related to its transcriptional activity.

CARM1 which locates at 19p13.2 is also known as the protein arginine methyltransferase 4 (PRMT4) [13]. It is reported that CARM1 acts as an oncogene in human cancers including ovarian, breast and lung cancers [16, 17, 29]. In present study, we also investigated the exact mechanism of PFKFB4-SRC-2 on LUAD progression. CARM1 was down-regulated extremely in SRC-2-knockdown LUAD cells by transcriptome screen. Knockdown of SRC-2 inhibited CARM1 expression while ectopic CARM1 co-overexpression could reverse the blockade. These findings indicated that CARM1 was transcriptionally regulated by SRC-2 in LUAD cells. What’s more, it is better to perform a luciferase reporter assay containing CARM1 promoter region to further verify the transcription activity of SRC-2. This work would be done in further research.

Our results showed that knockdown of PFKFB4 suppressed lung adenocarcinoma cells proliferation, migration and invasion could be restored by SRC-2 WT overexpression but not SRC-2 S487A, indicating that PFKFB4 promoted LUAD progression dependent on SRC-2 transcriptional activity. Phosphorylation of SRC-2 at S487 increased its transcriptional activity and upregulated CARM1 expression, thus promoting lung adenocarcinoma cells proliferation, migration and invasion. Taken together, our research uncovered the function and molecular mechanism of PFKFB4-SRC-2 signaling in LUAD progression, providing a new sight into therapy and treatment on LUAD.

## Supplementary Information

**Additional file 1. Figure S1**. SRC-2 overexpression could rescue the suppressed cell proliferation, migration and invasion induced by PFKFB4 knockdown. (A and B) A549 and NCI-H1975 were transfected with sh-PFKFB4 or sh-NC together with pCMV vector and/or SRC-2 WT/S487A. The expression of PFKFB4, SRC-2 and CARM1 at protein level was examined by western blot. (C and D) CCK-8 assay was performed among A549 and NCI-H1975 transfected with sh-PFKFB4 or sh-NC together with pCMV vector and/or SRC-2 WT/S487A. All experiments are performed in triple. *p < 0.05, **p < 0.01. (E and F) Transwell and Transwell-matrigel assay were performed among A549 and NCI-H1975 transfected with sh-PFKFB4 or sh-NC together with pCMV vector and/or SRC-2 WT/S487A. Representative images are captured with the magnification of ×200. **P < 0.01. All experiments are performed in triple.

## Data Availability

Gene expression analysis in lung adenocarcinoma tissues from TCGA samples was obtained from UALCAN (http://ualcan.path.uab.edu/).

## Abbreviations

PFKFB4: 6-Phosphofructo-2-kinase/fructose-2,6-biphosphatase-4
F2,6-BP: Fructose 2,6-bisphosphate
F6P: Fructose-6-phosphate
Pi: Inorganic phosphate
Etk: Endothelial tyrosine kinase
SCLC: Small-cell lung cancer
LUAD: Lung adenocarcinoma
CCK-8: Cell Counting Kit
