# Supplementary figures and images for: PFKFB4 promotes lung adenocarcinoma progression via phosphorylating and activating transcriptional coactivator SRC-2
Source: BMC Pulm Med. 2021 Feb 16;21:60. doi: 10.1186/s12890-021-01420-x (PMC7887818; doi:10.1186/s12890-021-01420-x)

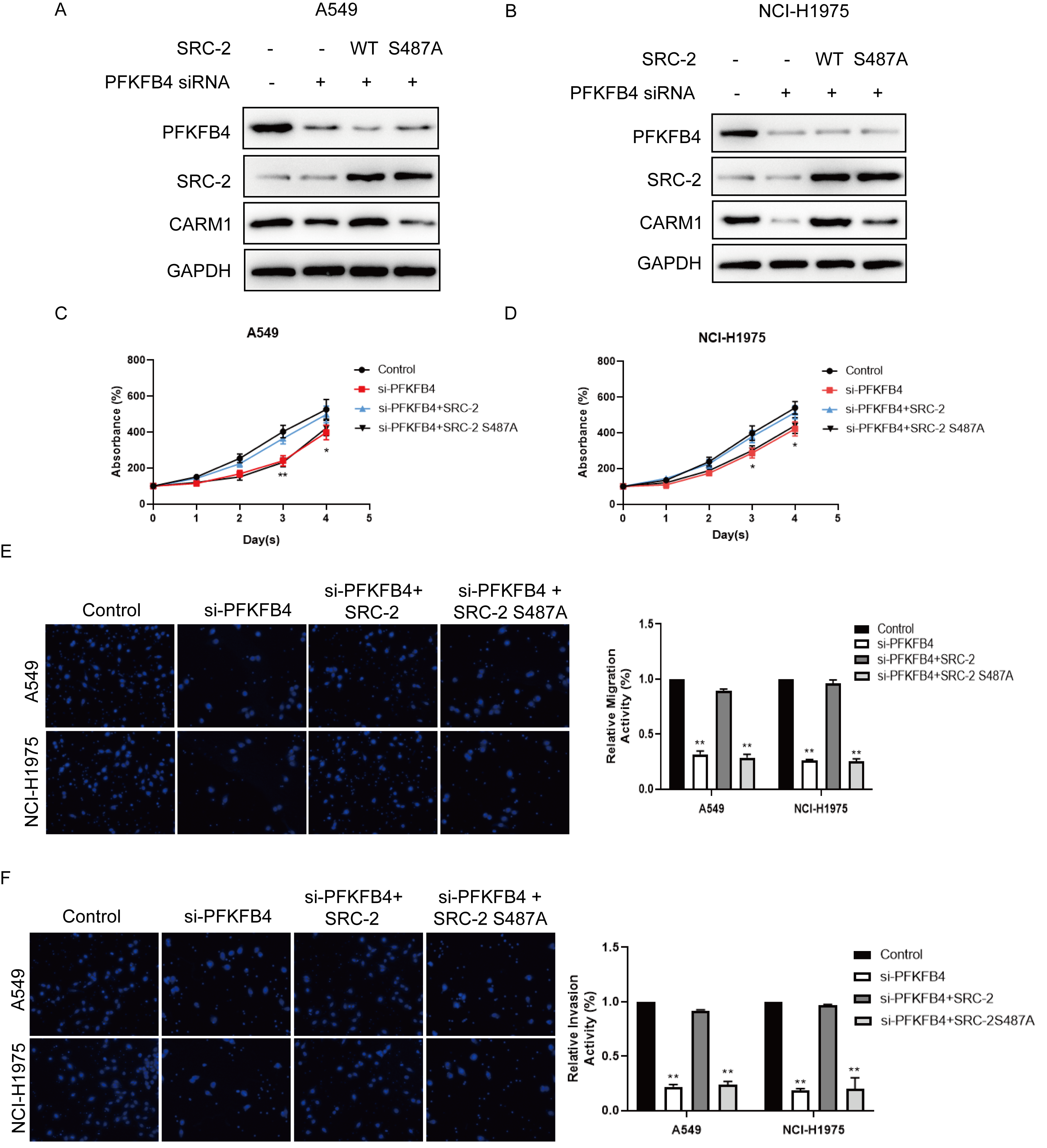

Supplement: Supplementary file 1 — Additional file 1. Figure S1. SRC-2 overexpression could rescue the suppressed cell proliferation, migration and invasion induced by PFKFB4 knockdown. (A and B) A549 and NCI-H1975 were transfected with sh-PFKFB4 or sh-NC together with pCMV vector and/or SRC-2 WT/S487A. The expression of PFKFB4, SRC-2 and CARM1 at protein level was examined by western blot. (C and D) CCK-8 assay was performed among A549 and NCI-H1975 transfected with sh-PFKFB4 or sh-NC together with pCMV vector and/or SRC-2 WT/S487A. All experiments are performed in triple. *p < 0.05, **p < 0.01. (E and F) Transwell and Transwell-matrigel assay were performed among A549 and NCI-H1975 transfected with sh-PFKFB4 or sh-NC together with pCMV vector and/or SRC-2 WT/S487A. Representative images are captured with the magnification of ×200. **P < 0.01. All experiments are performed in triple. [file 12890_2021_1420_MOESM1_ESM.tif]
